# LIFE COURSE EXAMINATION OF GLOBAL AND DAILY PSYCHOSOCIAL IMPACTS OF FAMILY CAREGIVERS IN MIDLIFE AND LATE ADULTHOOD

**DOI:** 10.1093/geroni/igz038.3020

**Published:** 2019-11-08

**Authors:** Jen D Wong, Jen D Wong, Yetty Shobo, Barbara T Hodgdon

**Affiliations:** 1 The Ohio State University, Columbus, Ohio, United States; 2 Virginia Board of Health Professions, Richmond, Virginia, United States

## Abstract

Family members often serve as informal caregivers for the first line of care. The complexity of family caregiving suggests the need to examine the personal and environmental resources that contribute to caregivers’ psychosocial well-being. Informed by the life course perspective, this study investigates the impacts of providing care to a family member on global and daily psychosocial well-being, and the moderating influences of age, gender, marital status, and social support. The sample consists of 1449 (M=55.99, SD=9.31) participants from Midlife in the United States (MIDUS-II: Main and Diary) survey. Regression and multilevel models results indicated greater global negative affect and daily stressors in caregivers as compared to non-caregivers. In line with the positive correlates of caregiving, caregivers reported greater daily positive events. Age, gender, and marital status significantly moderated the associations between caregiving and well-being. Findings showed that services aimed at family caregivers should take into account of personal resources.

